# A systematic genetic screen identifies new factors influencing centromeric heterochromatin integrity in fission yeast

**DOI:** 10.1186/s13059-014-0481-4

**Published:** 2014-10-02

**Authors:** Elizabeth H Bayne, Dominika A Bijos, Sharon A White, Flavia de Lima Alves, Juri Rappsilber, Robin C Allshire

**Affiliations:** Institute of Cell Biology, School of Biological Sciences, The University of Edinburgh, Edinburgh, EH9 3JR UK; Wellcome Trust Centre for Cell Biology, School of Biological Sciences, The University of Edinburgh, Edinburgh, EH9 3JR UK; Present address: Bristol Urological Institute, Southmead Hospital, Bristol, BS10 5NB UK

## Abstract

**Background:**

Heterochromatin plays important roles in the regulation and stability of eukaryotic genomes. Both heterochromatin components and pathways that promote heterochromatin assembly, including RNA interference, RNAi, are broadly conserved between the fission yeast *Schizosaccharomyces pombe* and humans. As a result, fission yeast has emerged as an important model system for dissecting mechanisms governing heterochromatin integrity. Thus far, over 50 proteins have been found to contribute to heterochromatin assembly at fission yeast centromeres. However, previous studies have not been exhaustive, and it is therefore likely that further factors remain to be identified.

**Results:**

To gain a more complete understanding of heterochromatin assembly pathways, we have performed a systematic genetic screen for factors required for centromeric heterochromatin integrity. In addition to known RNAi and chromatin modification components, we identified several proteins with previously undescribed roles in heterochromatin regulation. These included both known and newly characterised splicing-associated proteins, which are required for proper processing of centromeric transcripts by the RNAi pathway, and COP9 signalosome components Csn1 and Csn2, whose role in heterochromatin assembly can be explained at least in part by a role in the Ddb1-dependent degradation of the heterochromatin regulator Epe1.

**Conclusions:**

This work has revealed new factors involved in RNAi-directed heterochromatin assembly in fission yeast. Our findings support and extend previous observations that implicate components of the splicing machinery as a platform for RNAi, and demonstrate a novel role for the COP9 signalosome in heterochromatin regulation.

**Electronic supplementary material:**

The online version of this article (doi:10.1186/s13059-014-0481-4) contains supplementary material, which is available to authorized users.

## Background

Heterochromatin is a condensed form of chromatin of fundamental importance to the regulation and stability of eukaryotic genomes. It is characterised by methylation of histone H3 on lysine 9, a specific chromatin signature that facilitates binding of chromodomain proteins and other factors to create a transcriptionally repressive chromatin state [1]. Evidence from several systems indicates that non-coding RNAs can play important roles in attracting chromatin modifiers to target loci [2]. In particular, small RNAs generated by the RNA interference (RNAi) pathway can direct nucleation of heterochromatin domains that can be further propagated via spreading in cis [3,4]. The molecular mechanisms underpinning the targeting and regulation of RNAi-directed heterochromatin formation are still not well understood, but are arguably best characterised in the fission yeast *Schizosaccharomyces pombe*, which possesses relatively simple but conserved RNAi and chromatin modification pathways, making it a powerful model system for dissecting mechanistic principles of eukaryotic heterochromatin assembly.

In fission yeast, domains of constitutive heterochromatin are found at centromeres, telomeres, and the silent-mating-type locus. H3K9 methylation is mediated by a sole H3K9 methyltransferase, Clr4, which can be recruited to chromatin via both RNAi-dependent and independent pathways [1]. At centromeres, the RNAi machinery is important for both establishment and maintenance of heterochromatin. Although heterochromatic, centromeric outer repeat sequences are transcribed during S phase by RNAPII, generating double-stranded (ds) RNA that is processed into short-interfering (si) RNAs by Dicer (Dcr1) [3,4]. These siRNAs guide the Argonaute (Ago1) containing RITS complex to homologous nascent transcripts, resulting in recruitment of further factors including the RNA-dependent RNA polymerase complex (RDRC) that amplifies the RNAi signal. The RDRC component Cid12 also interacts with components of the splicing machinery, which are implicated in promoting processing of heterochromatic transcripts by the RNAi pathway [5]. Ultimately, transcript-bound RITS leads to the recruitment of the H3K9 methyltransferase Clr4, mediated by the bridging protein Stc1 [6,7]. Methylation of H3K9 creates binding sites for chromodomain proteins including the HP1-related proteins Swi6 and Chp2, as well as Clr4 and the RITS component Chp1, so that siRNA generation and chromatin modification form a self-reinforcing loop [8-10]. RNAi also contributes to heterochromatin assembly at the telomeres and silent mating-type locus, targeting defined sequence elements with homology to centromeric outer repeats. However, here, RNAi is required only for establishment but not maintenance of heterochromatin, since alternative pathways act redundantly with RNAi to facilitate ongoing recruitment of chromatin modifiers to these regions [11-13]. Recent evidence suggests that alternative, RNAi-independent pathways can also promote heterochromatin assembly at centromeres, although the mechanisms and significance of these are as yet unclear [14-16]. Moreover, a proportion of H3K9 methylation is maintained in the absence of RNAi, and this has been shown to be dependent on the histone deacetylases (HDACs) Sir2 and Clr3/SHREC [17,18].

The H3K9 methyltransferase Clr4 is found within a multi-protein complex called CLRC, all members of which are required for heterochromatin assembly [19-22]. In addition to Clr4, CLRC comprises the Cullin protein Cul4, Rik1, Raf1 and Raf2. Cullins function as scaffold proteins within conserved Cullin-RING ubiquitin ligase (CRL) complexes [23]. The activity of these complexes is regulated via neddylation of the Cullin subunit, which in turn is regulated by components of the COP9 signalosome complex [24]. The CLRC component Rik1 is very similar to DDB1, which is a specific adapter protein of Cul4 CRLs, while Raf1 resembles a CRL substrate specificity factor (DCAF). The fission yeast Cul4-Rik1^Raf1^ complex is therefore thought to represent a specialised paralog of the canonical Cul4-Ddb1^DCAF^ complex, in which Rik1 and Raf1 substitute for Ddb1 and the DCAF, respectively [25,26]. Consistent with this, purified CLRC exhibits E3 ubiquitin ligase *in vitro* [20], and mutation of the Cul4 neddylation site prevents H3K9 methylation *in vivo* [21], although *in vivo* ubiquitination substrates have not yet been identified. Interestingly, in addition to the essential role of CLRC in H3K9 methylation, a role in maintaining robust heterochromatin has also recently been uncovered for the canonical Cul4-Ddb1^DCAF^ complex [27]. Deletion of either Ddb1, or the DCAF Cdt2, causes a modest defect in heterochromatin, associated with increased accumulation of Epe1 within heterochromatic domains. Although the precise function of Epe1 is unclear, it appears to antagonise heterochromatin formation, in particular suppressing the invasion of heterochromatin into euchromatic domains [28-30]. Heterochromatin defects in *ddb1Δ* mutant cells are largely alleviated by deletion of Epe1, consistent with a model in which Cul4-Ddb1^Cdt2^ contributes to the integrity of heterochromatin by mediating the ubiquitination, and hence degradation, of Epe1 bound within the interior of heterochromatin domains [27].

Rapid progress in the identification of factors required for heterochromatin assembly in fission yeast has been made through a combination of genetic and biochemical approaches. The use of reporter genes to monitor heterochromatin integrity has proved a particularly powerful tool: because genes embedded in heterochromatin are typically repressed or ‘silenced’ (a phenomenon termed position effect variegation), loss of silencing represents a convenient indicator of defective heterochromatin [31]. Previous genetic screens employing random mutagenesis in combination with this type of assay identified key pathway components such as Clr4, as well as accessory factors including splicing factors [5,32,33]. However, these screens were hindered by difficulties in identifying causative mutations, and did not reach saturation. More recently, small-scale systematic screens, employing candidate approaches based on published protein localisation data, have identified further factors impacting on the pathway [27,34,35]. However, a systematic genome-wide analysis has not yet been reported. Here we describe just such a genome-wide genetic screen to identify all non-essential fission yeast proteins required for centromeric heterochromatin formation. This screen identified the majority of components with known roles in heterochromatin formation, plus Stc1, a novel factor critical to the pathway and described elsewhere [6]. In addition, the screen uncovered several additional accessory factors required for robust heterochromatic silencing. These include two components of the COP9 signalosome, Csn1 and Csn2, as well as four proteins with functional links to splicing. The findings shed new light on the regulation of heterochromatin assembly as well as its integration with other cellular pathways, and provide a more complete understanding of the non-essential factors required for RNAi-directed heterochromatin formation in fission yeast.

## Results

### A systematic screen for factors required for centromeric heterochromatin integrity

**Figure 1:**
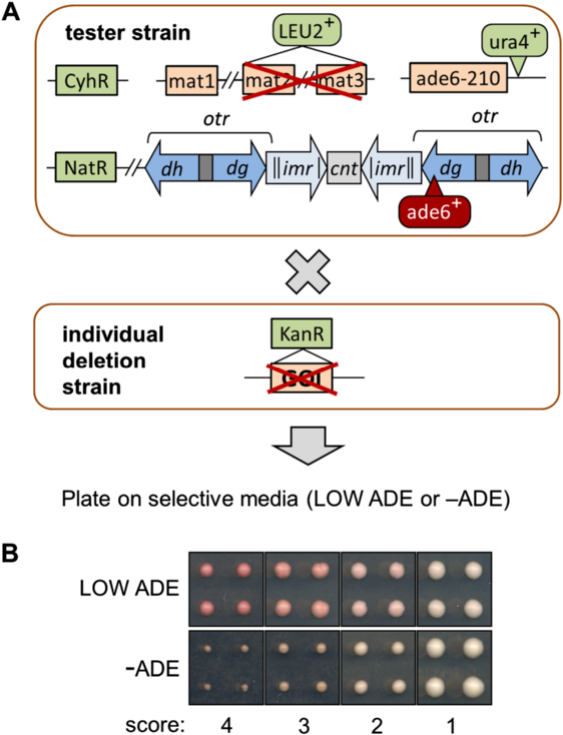
**Genetic screen for mutants defective in heterochromatic silencing. (A)** Schematic representation of the screening strategy. High-throughput crossing of a gene deletion library to a tester strain (see text for further details) facilitated analysis of the effects of individual gene deletions on silencing of an *ade6* ^*+*^ reporter gene inserted into the centromeric outer repeats of centromere 1 (*cen1:ade6* ^+^; *otr* (*dg* and *dh*): outer repeats; *imr*: innermost repeats; *cnt*: central core) **(B)** Illustration of the semi-quantitative system used to score defects in *cen1:ade6* ^*+*^ silencing, based on colony colour on plates containing limiting levels of adenine (LOW ADE) or colony size on plates lacking adenine (-ADE), where 4 is equivalent to wild-type silencing, and 1 indicates strong *ade6* ^*+*^ de-repression.

We utilised a haploid gene deletion set [36] to systematically screen for factors contributing to centromeric heterochromatin integrity in fission yeast. A tester strain was created bearing an *ade6*^*+*^ reporter gene inserted into the heterochromatic outer repeats of centromere 1 (*cen1:ade6*^*+*^, Figure 1A). Normally, the presence of heterochromatin silences the inserted *ade6*^*+*^ gene; *ade6*^*+*^ expression is therefore an indicator of defects in heterochromatin integrity. A nourseothricin resistance cassette (NatR) inserted close to centromere 1 allowed selection for *cen1:ade6*^*+*^, while the *ade6-210* mutant allele at the endogenous *ade6*^*+*^ locus was selected via an adjacent *ura4*^*+*^ cassette. The tester strain also contained the P56Q allele of the ribosomal protein gene *rpl42*^+^, which confers robust and recessive resistance to cyclohexamide thereby providing a means of selecting against diploids [37], as well as a deletion of the silent mating-type loci, *mat2(P)* and *mat3(M)*, marked with *LEU2*^+^, to allow selection for homogeneity in mating-type. High throughput crossing of the tester strain to the deletion library (384-well format, with each deletion mutant in quadruplicate) followed by direct plating on selective media allowed us to select haploid cells bearing both a single gene deletion and the *cen1:ade6*^*+*^ reporter gene (Figure 1A). *cen1:ade6*^*+*^ expression was then assessed in two ways: via colour on media containing limiting adenine (low ade; red indicates *ade6*^*+*^ silencing, pink/white indicates *ade6*^*+*^ expression) and via growth on media lacking supplementary adenine (-ade; growth indicates *ade6*^*+*^ expression). Scoring was done semi-quantitatively on a scale of 1 to 4, where 4 is equivalent to wild-type silencing, and 1 indicates strong *ade6*^*+*^ de-repression (Figure 1B).

According to PomBase [38], a total of 53 *S. pombe* genes have thus far been annotated as being involved in chromatin silencing at centromeres, of which 28 were represented in the deletion set. As expected, the majority of top hits identified in the screen belonged to this group of ‘known’ genes, validating the approach (Additional file 1: Table S1 and Additional file 2). In particular, the scoring of these mutants indicated that growth on -ade plates was the most effective predictor of heterochromatin factors: known genes (including Stc1, which was identified in this screen and published previously [6]) represented 13 out of 17 mutants with a growth score of 1, plus six out of 16 mutants with a growth score of 2. In contrast, when assessed by colour on low ade plates, known centromere silencing factors represented 13 out of 18 mutants with a colour score of 1, but only four out of 43 mutants with a colour score of 2. Thus growth on media lacking adenine was the most efficient predictor of a greater number of known components. No known factors scored 3 for growth on -ade; we therefore set a cutoff of a growth score of 2 to generate a shortlist of candidate mutants for further analysis. By this criterion, 19 known components were identified in the screen, including all core RNAi components (Dcr1, ARC, RITS and CLRC) except Ago1 and Hrr1 (Additional file 1: Table S1). Importantly, several factors whose deletion is known to have only modest effects on silencing at this locus, including the HDAC Sir2, were also represented in the shortlist, demonstrating the sensitivity of our approach. Nine genes annotated as involved in centromere silencing were present in the library but absent from the shortlist. For three of these genes, including *ago1*^*+*^ and *hrr1*^*+*^, PCR analysis of the corresponding deletion strain revealed that the target ORF was in fact still present, explaining the lack of phenotype. Other genes that were correctly deleted but not identified in the screen included SHREC complex components *clr1*^*+*^, *clr2*^*+*^, *clr3*^*+*^ and *mit1*^*+*^ [39] - it appears that effects of these mutants on silencing at the particular centromeric locus analysed were too weak to detect in this assay.

**Figure 2:**
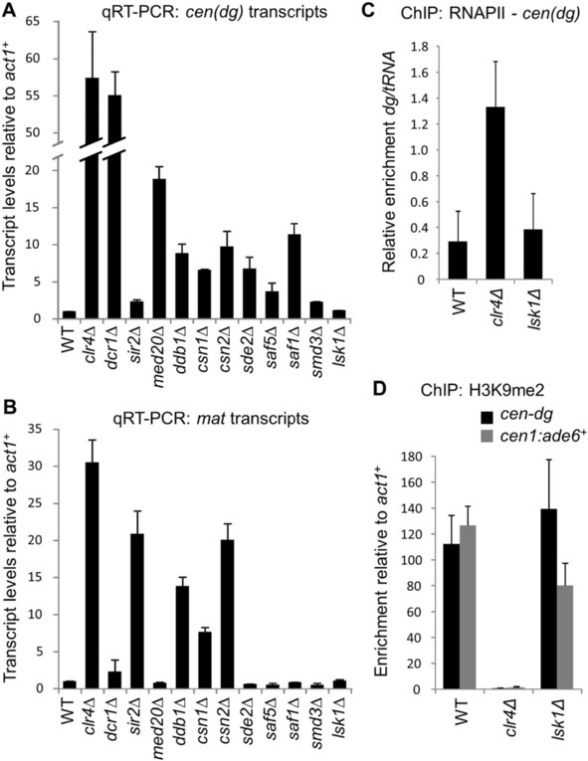
**The majority of screen hits cause de-repression at centromeric outer repeats. (A, B)** qRT-PCR analysis of centromeric outer repeat (A; *cen-dg*) and mating-type locus (B; *mat*) transcript levels relative to *act1* ^*+*^, normalised to wild-type. Along with *clr4Δ* and *dcr1Δ*, *sir2Δ* was included as a control for the sensitivity of the assay since this mutant is known to cause only mild de-repression at the centromeric locus analysed. **(C)** ChIP analysis of RNAPII levels at the centromeric outer repeats (*cen-dg*) relative to tRNA in *lsk1Δ* mutant cells. **(D)** ChIP analysis of H3K9me2 levels at the centromeric outer repeats (*cen-dg*) and *cen1:ade6* ^*+*^ reporter gene relative to the *act1* ^*+*^ gene in *lsk1Δ* mutant cells.

The shortlist contained 14 mutants without annotated roles in heterochromatin assembly (Additional file 1: Table S2). In two of these mutants the designated gene proved not to be deleted based on PCR analysis, and these strains were excluded. For the remaining 12 mutants, to verify that the apparent increase in expression of the *cen1:ade6*^*+*^ reporter reflected de-repression of endogenous centromeric sequences, we directly tested accumulation of non-coding centromeric outer repeat transcripts by qRT-PCR. To ascertain whether the effect was specific to the centromere, transcripts from another heterochromatic region, the silent mating-type locus, were also analysed. Five of the mutants exhibited no effects on endogenous heterochromatic transcript accumulation (Figure 2 and Additional file 1: Table S2). To further investigate these five mutants, we re-tested silencing using a *ura4*^*+*^ reporter gene instead of an *ade6*^*+*^ reporter gene at the same centromeric locus. For four of the mutants, no effects on *cen1:ura4*^*+*^ silencing were observed (Additional file 1: Figure S1). Since the effects of these mutants on silencing were not reproduced on a second reporter gene they were considered false positives and disregarded. One of the mutants, a deletion of the nuclear kinase Lsk1, did exhibit de-repression of *cen1:ura4*^*+*^, as indicated by increased growth on plates lacking uracil (Additional file 1: Figure S1). This mutant therefore appears to have a general effect on heterochromatic reporter gene silencing. It was possible that in this mutant centromeric silencing is disrupted without any observable increase in centromeric transcript accumulation if, for example, transcription is impaired or the transcripts rapidly degraded. However, ChIP analysis did not detect any increase in RNAPII on centromeric repeats in the *lsk1Δ* mutant, arguing against the latter possibility (Figure 2C). Moreover, although a partial reduction in H3K9 methylation was observed on the cen1:*ade6*^*+*^ reporter gene, levels of H3K9 methylation on the endogenous centromeric repeats were unaffected in the absence of Lsk1 (Figure 2D). Since the *lsk1Δ* mutant appeared to specifically affect silencing of embedded marker genes but not endogenous centromeric repeats, it was not analysed further.

Seven mutants were confirmed to disrupt silencing within the centromeric outer repeats, accumulating levels of centromeric transcripts at least two-fold higher than those seen in wild-type cells, and similar or higher than those seen in cells lacking the HDAC Sir2 (Figure 2A). Among these mutants were deletions of two proteins that, although not annotated in PomBase as affecting chromatin silencing at centromeres, have previously been reported to have a modest impact on centromeric heterochromatin integrity: the mediator subunit Med20 [40], and CRL adaptor protein Ddb1 [27]. Aside from these two mutants whose centromeric silencing defects have already been characterised, we identified five mutants with no previously described effects on heterochromatin silencing. These mutants could be further subdivided into two classes based on their effects at different heterochromatic loci: three affecting specifically centromeric silencing (*smd3Δ*, *saf1Δ* and *SPAC1610.01/saf5Δ*), and two affecting both the centromere and silent mating-type locus (*csn1Δ* and *csn2Δ*, Figure 2A and B). For each of these genes we generated new, independent deletion strains for further analysis.

### Deletion of splicing-associated factors affects RNAi-dependent heterochromatin integrity

**Figure 3:**
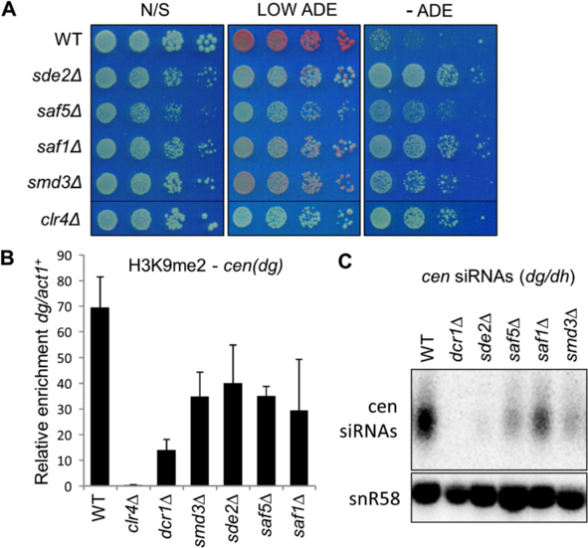
**Sde2, Smd3, Saf1 and Saf5 affect RNAi-dependent heterochromatin formation. (A)** *cen1:ade6* ^*+*^ silencing assay. Equivalent cell numbers of the indicated strains were spotted in serial dilutions on non-selective plates (N/S), or plates containing 10 ug/mL adenine (LOW ADE) or no adenine (-ADE). **(B)** ChIP analysis of H3K9me2 levels at the centromeric outer repeats (*cen-dg*) relative to the *act1* ^*+*^ gene, normalised to wild-type. **(C)** Northern analysis of centromeric siRNAs. snoRNA58 (snR58) is a loading control.

We first investigated the genes found to specifically affect centromeric silencing. These included Smd3, a core snRNP protein involved in splicing [41], Saf1, a protein found to co-purify with components of the splicing machinery [42], and a conserved but poorly characterised gene *SPAC1610.01*, which we refer to as Saf5 (see below). Sde2, a protein recently implicated in heterochromatic silencing particularly at telomeres [35], was also included in this analysis as its role in centromeric silencing was unannotated at the time, and is still not well characterised. All four mutants exhibit defects in *cen1:ade6*^*+*^ marker gene silencing that are detectable via both pale colour on plates containing limiting adenine, and increased growth in the absence of adenine (Figure 3A). Moreover, all of the mutants also exhibit reduced levels of centromeric H3K9 methylation by ChIP, confirming that the observed loss of silencing reflects defective heterochromatin (Figure 3B). RT-PCR analysis indicated that these mutants cause de-repression at the centromere but not the silent mating-type locus (Figure 2A and B), and this was confirmed by silencing assays which showed no effects of these mutants on expression of a *ura4*^*+*^ reporter gene inserted into the mating-type locus (Additional file 1: Figure S2). Since the RNAi pathway is required for maintenance of heterochromatin specifically at the centromere and not the mating-type locus, this suggests that these mutants impact on heterochromatin formation at the level of the RNAi pathway. Consistent with this, northern analysis revealed reduced accumulation of centromeric siRNAs in all the mutants, indicative of defective RNAi-mediated processing of non-coding centromeric transcripts (Figure 3C).

**Figure 4:**
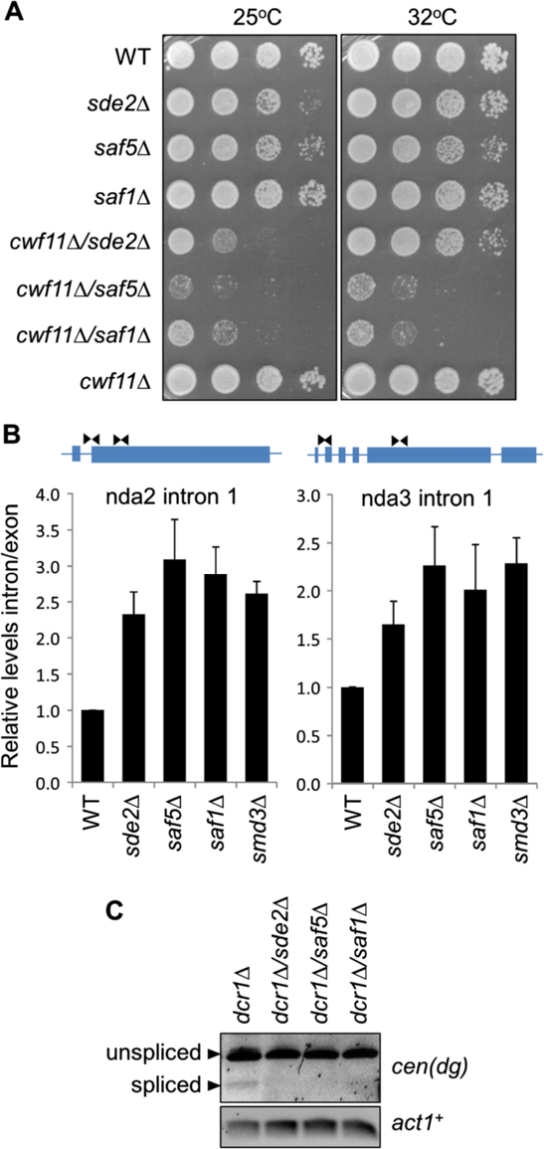
**Sde2 and Saf5 are involved in splicing. (A)** Synthetic interaction with *cwf11∆*. Equivalent cell numbers of the indicated strains were spotted in serial dilutions and incubated at 32°C for 3 days, or 25°C for 4 days. **(B)** Splicing assay. Accumulation of intron relative to exon RNA in wild-type versus mutant strains was measured for *nda2* ^*+*^ and *nda3* ^*+*^ by qRT-PCR analysis using primer pairs either spanning an intron-exon boundary or within an exon, as illustrated above. **(C)** Splicing of centromeric outer repeat (*cen-dg*) non-coding transcripts. Accumulation of spliced and unspliced *cen(dg)* transcripts were monitored by RT-PCR using primers flanking a previously reported intron sequence.

Both Smd3 and Saf1 are functionally linked to splicing [41,42]. To gain further insight into the molecular function of Saf5 and Sde2, we epitope-tagged each protein at the endogenous locus, affinity purified it from cell lysates, and identified co-precipitating proteins by liquid chromatography - tandem mass spectrometry (LC-MS/MS). Saf5 was found to specifically associate with components of the splicing machinery, most notably, all components of the core snRNP including Smd3 (Table 1). Consistent with this finding, this gene has also recently been implicated in splicing by epistasis mapping [43]. Given this functional link to splicing, and following established nomenclature for splicing-associated proteins [42], we named the product of this ORF Saf5, for Splicing-Associated Factor 5. Although Sde2 has been reported to contribute to heterochromatic silencing, its molecular function remains unknown [35]. Strikingly, our analysis revealed that Sde2 also co-purifies with a wide range of splicing factors, suggesting it too is associated with splicing (Table 2). In support of this, we note that Sde2 was also previously detected among interactors of the splicing factors Prp17 and Prp19 [42]. To provide further evidence of a functional role for these proteins in splicing, we tested for synthetic interactions with the known splicing mutant *cwf11Δ. saf1Δ*, *saf5Δ* and *sde2Δ* all displayed negative genetic interactions *cwf11Δ*, consistent with links to splicing (Figure 4A). These mutants also displayed synthetic interactions with the temperature-sensitive splicing mutants *cdc5-120* and *prp1-1* (Additional file 1: Figure S3). Moreover, qRT-PCR analysis revealed that, like cells lacking the known splicing factor Smd3, cells bearing deletions of Saf1, Saf5 or Sde2 all accumulate elevated levels of un-spliced transcripts in comparison with wild-type cells, confirming a role for these proteins in the splicing pathway (Figure 4B).

**Table 1:** **Proteins associated with Saf5**

**Systematic ID**	**Gene name**	**Peptide count**	**Mol weight (kDa)**
SPBC19C2.14	*smd3*	7.0	11.0
SPAC26A3.08	*smb1*	6.3	15.5
**SPAC1610.01**	***saf5***	**6.3**	**25.0**
SPAC27D7.07c	*smd1*	6.0	13.1
SPAC2C4.03c	*smd2*	6.0	13.1
SPAC2G11.08c	*smn1*	5.0	17.4
SPAC4F8.12c	*spp42*	4.7	274.6
SPBC11G11.06c	*sme1*	3.7	9.7
SPBC16H5.15		3.0	19.7
SPAPB17E12.02	*yip12*	3.0	27.0
SPBC4B4.05	*smg1*	2.7	8.6
SPBC3E7.14	*smf1*	2.3	8.7
SPAC644.12	*cdc5*	2.3	86.8
SPAC29A4.08c	*prp19*	2.0	54.2
SPBC31F10.11c	*cwf4*	2.0	80.8
SPBC215.12	*cwf10*	1.3	111.3

List of all proteins identified by mass spectrometry in three independent affinity purifications of Saf5-FLAG, and absent from control purifications. Peptide counts represent average numbers of peptides identified across the three replicates. The bait protein is highlighted in bold.

**Table 2:** **Proteins associated with Sde2**

**Systematic ID**	**Gene name**	**Peptide count**	**Mol weight (kDa)**
SPAC4F8.12c	*spp42*	123.3	274.6
SPAC9.03c	*brr2*	63.0	248.8
SPBC646.02	*cwf11*	46.7	148.4
SPBC215.12	*cwf10*	44.7	111.3
SPAC644.12	*cdc5*	43.0	86.8
SPBC211.02c	*cwf3*	40.7	92.6
SPAC30D11.09	*cwf19*	35.0	74.4
SPBC31F10.11c	*cwf4*	32.3	80.8
SPBC6B1.10	*prp17*	31.7	63.1
SPAC10F6.02c	*prp22*	31.7	131.5
SPCC188.11	*cwf13*	28.7	62.7
SPBC13E7.01	*cwf22*	25.0	102.7
SPBP22H7.07	*prp5*	23.0	52.4
SPAC3A12.11c	*cwf2*	19.3	44.3
SPCC550.02c	*cwf5*	17.3	39.6
SPBC1289.11	*spf38*	16.3	37.4
SPAC29A4.08c	*prp19*	15.0	54.2
SPBC3E7.13c	*syf2*	14.7	28.0
SPBC337.06c	*cwf15*	14.7	30.4
SPBC32F12.05c	*cwf12*	13.7	25.6
SPBC28F2.04c	*cwf7*	12.3	21.3
**SPAC31G5.18c**	***sde2***	**11.0**	**29.2**
SPCP1E11.07c	*cwf18*	9.0	16.7
SPBC24C6.11	*cwf14*	8.3	17.1
SPBC530.14c	*dsk1*	8.3	61.1
SPAC4A8.09c	*cwf21*	7.3	34.6
SPBC18H10.10c	*saf4*	6.3	34.7
SPAC16.02c	*srp2*	6.0	42.6
SPAC26A3.08	*smb1*	3.3	15.5
SPAC27F1.09c	*prp10*	3.3	132.7
SPBC146.05c	*cwf25*	3.0	46.1
SPCC1620.10	*cwf26*	2.3	36.0
SPAPJ698.03c	*prp12*	2.3	135.0
SPBC11C11.08	*srp1*	2.0	31.1

List of all proteins identified by mass spectrometry in three independent affinity purifications of Sde2-FLAG, and absent from control purifications. Peptide counts represent average numbers of peptides identified across the three replicates. The bait protein is highlighted in bold.

We previously reported that specific temperature-sensitive splicing mutants impair RNAi-dependent heterochromatin assembly in fission yeast, independently of their role in splicing, likely reflecting a requirement for splicing factors to provide a platform for RNAi recruitment and/or processing [5]. Given the similarity in phenotypes between the previously described splicing mutants and those identified here, it is very likely that they impact on heterochromatin via the same mechanism. Consistent with this, replacement of genomic copies of two key RNAi genes that contain introns, *ago1*^*+*^ and *hrr1*^*+*^, with intron-less versions failed to rescue the silencing defects in the newly identified splicing mutants, indicating that the observed defects in silencing cannot be explained by impaired splicing of these pathway components (Additional file 1: Figure S4). While we cannot exclude that defective splicing of some other contributory factor might be involved, we instead sought further evidence of a direct role of splicing components in processing of centromeric transcripts. Two previous studies have reported evidence of splicing of centromeric transcripts [44,45]. To confirm this and investigate the sensitivity of these splicing events to splicing mutants, we analysed centromeric transcripts by RT-PCR. The analyses were performed in a *dcr1Δ* deletion background, since this induces accumulation of high levels of centromeric transcripts, facilitating their analysis. Consistent with previous reports, we were able to detect a shorter, spliced form of *cen-dg* transcript in addition to the primary RNA (Figure 4C). That this represented a bona fide splice product was confirmed by sequencing (Additional file 1: Figure S5). Moreover, deletion of the splicing-associated factors Sde2, Saf1 or Saf5 resulted in greatly reduced accumulation of the spliced form of the *cen-dg* RNA (Figure 4C). This finding indicates that these factors are directly involved in the processing of centromeric non-coding RNAs, and is consistent with a model in which the direct activity of splicing factors on centromeric transcripts somehow facilitates their processing by the RNAi machinery.

### Deletion of Csn1 or Csn2 affects heterochromatin integrity independently of RNAi

**Figure 5:**
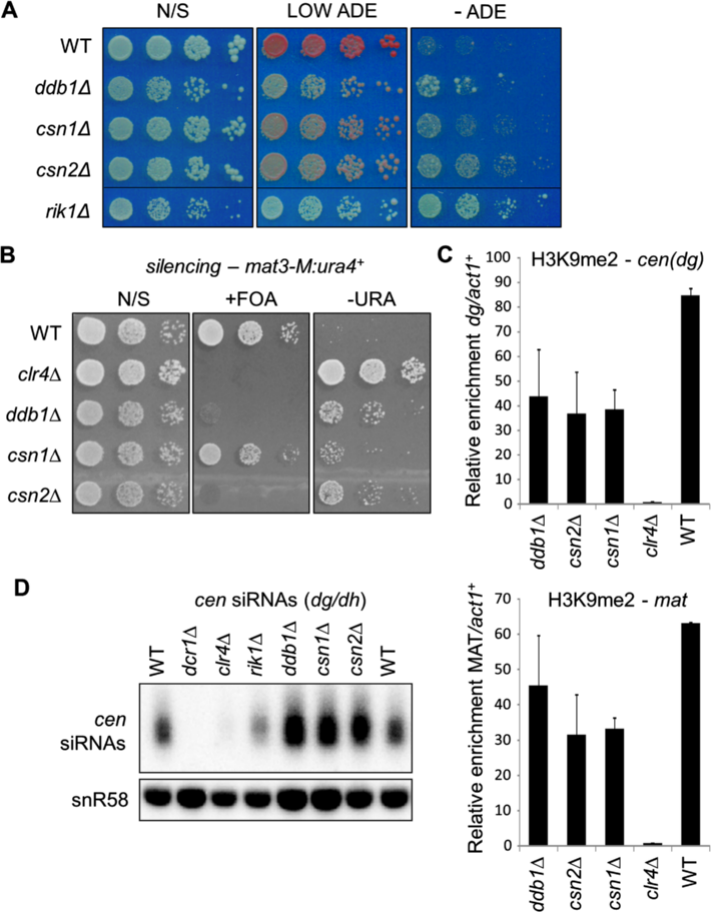
**Csn1 and Csn2 affect heterochromatin integrity independently of RNAi. (A)** *cen1:ade6* ^*+*^ silencing assay. Equivalent cell numbers of the indicated strains were spotted in serial dilutions on non-selective plates (N/S), or plates containing 10ug/ml adenine (LOW ADE) or no adenine (-ADE). **(B)** Assay for silencing at the silent mating-type locus (*mat3-M:ura4* ^*+*^). Plates are non-selective (N/S), lacking uracil (-URA) or supplemented with FOA (+FOA). **(C)** ChIP analysis of H3K9me2 levels at centromeric outer repeats (*cen-dg*) or the silent mating-type locus (*mat*) relative to *act1* ^*+*^, normalised to wild-type. **(D)** Northern analysis of pericentromeric siRNAs. snoRNA58 (snR58) is a loading control.

We next investigated the mutants that were found to disrupt silencing at both the centromere and silent mating-type locus: *csn1Δ* and *csn2Δ*. Csn1 and Csn2 are components of the COP9 signalosome, which is involved in regulating cullin-dependent E3 ubiquitin ligases [23,24]. The finding that deletion of these proteins impacts on heterochromatin was striking since two distinct Cul4 complexes are implicated in the establishment and maintenance of heterochromatin domains: the Clr4 complex CLRC (Clr4/Cul4/Rik1/Raf1/Raf2), and the related E3 ubiquitin ligase complex Cul4-Ddb1^Cdt2^. We therefore compared cells lacking Csn1 or Csn2 to those lacking the CLRC component Rik1, or paralogous Cul4-Ddb1^Cdt2^ component Ddb1. Silencing assays revealed that *csn1Δ* and *csn2Δ* mutant cells exhibit defects in *cen1:ade6*^*+*^ marker gene silencing that are similar to those in *ddb1Δ* mutant cells, but milder than those in *rik1Δ* mutant cells (Figure 5A). This mirrors the pattern observed in qRT-PCR analysis of endogenous centromeric transcript levels (Figure 2A). Transcript analysis also indicated that loss of Csn1 or Csn2 additionally causes de-repression at the mating-type locus, and this was confirmed by a reporter gene silencing assay: heterochromatic silencing of *ura4*^*+*^ inserted into the silent mating type locus (*mat3-M:ura4*^*+*^) is disrupted upon deletion of Ddb1, Csn2 or (to a lesser extent) Csn1, as evidenced by increased growth on media lacking uracil, and reduced growth in the presence of the counter-selective drug FOA (Figure 5B). Moreover, ChIP analysis revealed that cells lacking Csn1 or Csn2 also display reduced H3K9 methylation at both the centromere and mating-type locus (Figure 5C). The reduction in H3K9 methylation in *csn1Δ* and *csn2Δ* cells is similar to that seen in *ddb1Δ* cells, but modest compared to the complete loss of methylation that occurs in *clr4Δ* cells, indicating that Csn1 and Csn2 are not required for the H3K9 methyltransferase activity of CLRC. The finding that Csn1 and Csn2 impact on maintenance of heterochromatin at the silent mating-type locus, which is RNAi-independent, does however suggest that these factors contribute to heterochromatin assembly at the level of chromatin modification, rather than RNAi. To rule out any impact on the RNAi pathway, we also assessed levels of centromeric siRNAs by northern analysis. Whereas deletion of Clr4 or Rik1 cause a decrease in siRNA accumulation, no reduction is seen upon deletion of Ddb1, Csn1 or Csn2; in fact, siRNA levels are slightly elevated in these cells, likely due to the increased levels of non-coding centromeric transcripts available for processing by the RNAi pathway (Figure 5D). Thus deletion of Csn1 or Csn2 does not impair the RNAi pathway, and must therefore impact heterochromatin via downstream chromatin modifier(s).

### Csn1 and Csn2 are required for regulation of Epe1

**Figure 6:**
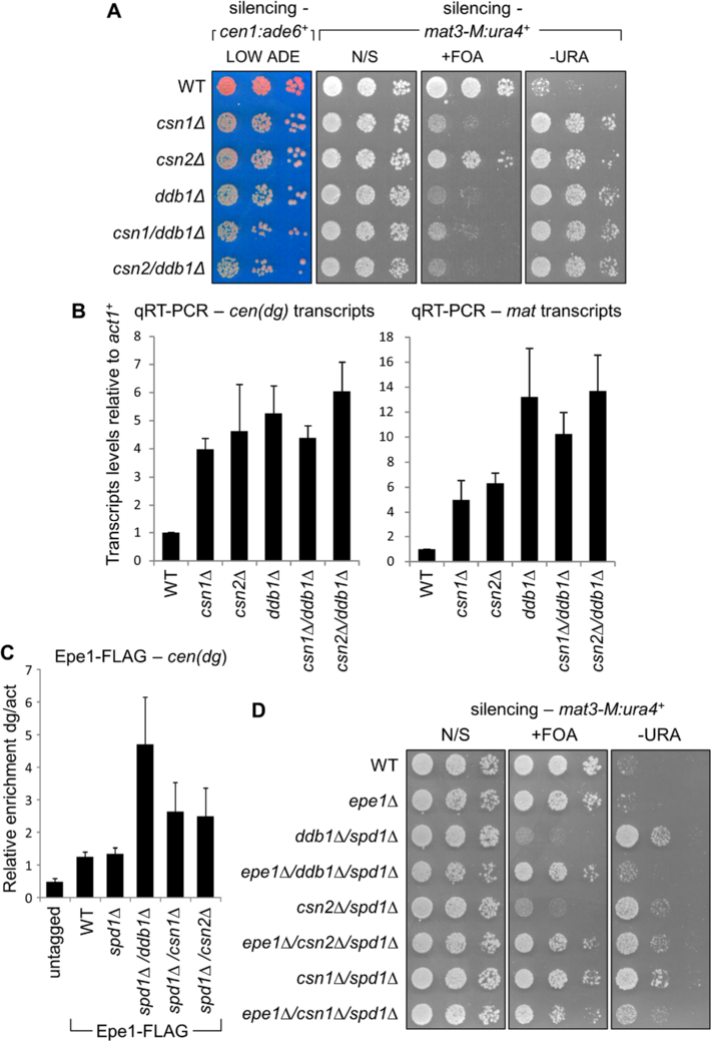
**Csn1 and Csn2 contribute to heterochromatin integrity via regulation of Epe1. (A, B)** Analysis of genetic interactions of *csn1Δ* and *csn2Δ* with *ddb1Δ*. **(A)** Assays for silencing of reporter genes at the centromere (*cen1:ade6* ^*+*^ *)* and mating-type locus (*mat3-M:ura4* ^*+*^ *).* Plates are limiting adenine (LOW ADE), non-selective (N/S), lacking uracil (-URA) or supplemented with FOA (+FOA). **(B)** qRT-PCR analysis of centromeric outer repeat (*cen-dg*) and mating-type locus (*mat*) transcript levels relative to *act1* ^*+*^, normalised to wild-type. **(C)** ChIP analysis of Epe1-FLAG levels at centromeric outer repeats (*cen-dg*) relative to *act1* ^*+*^, normalised to wild-type. **(D)** Assay for silencing at the mating-type locus (*mat3-M:ura4* ^*+*^).

The phenotypes of cells lacking Csn1 or Csn2 closely resemble those of cells lacking Ddb1. To assess whether Csn1/Csn2 and Ddb1 might function in the same pathway, we generated *csn1Δ/ddb1Δ* and *csn2Δ/ddb1Δ* double mutants. Silencing assays and qRT-PCR analysis revealed that silencing at both the centromere and silent mating-type locus is impaired to a similar extent in the double mutants as it is in the *ddb1Δ* single mutant (Figure 6A and B); that the effects of these mutants on silencing are non-additive is consistent with them acting in the same pathway. The Cul4-Ddb1^Cdt2^ complex is implicated in the regulation of at least two substrate proteins: Spd1, an inhibitor of ribonucleotide reductase, and Epe1, a heterochromatin regulator [27,46]. Spd1 and Epe1 hyper-accumulate in cells lacking Ddb1, and the resulting defects in cell cycle and heterochromatic silencing can be largely rescued by deletion of Spd1 and Epe1, respectively. Since Csn1 and Csn2 have been shown to function alongside Ddb1 and Cul4 in the regulation of Spd1 [46-48], we hypothesised that they may contribute to heterochromatin integrity via a similar role in regulation of Epe1. To test this we first analysed accumulation of FLAG-tagged Epe1 within heterochromatic domains by ChIP. Consistent with previous findings, we detected elevated levels of Epe1 on centromeric outer repeat sequences in *ddb1Δ*/*spd1Δ* double mutant cells (Figure 6C; the *spd1Δ* deletion background was used for these experiments to exclude any indirect effects of cell cycle-related growth defects). Interestingly, levels of Epe1 were also found to be elevated in *csn1Δ*/*spd1Δ* and *csn2Δ*/*spd1Δ* mutant cells, although to a lesser extent than in *ddb1Δ*/*spd1Δ* cells; this is consistent with a model in which deletion of Csn1 or Csn2 impairs the function of the Cul4-Ddb1^Cdt2^ complex in removal of Epe1 from heterochromatin. We also tested whether deletion of Epe1 can rescue the heterochromatic silencing defect in *csn1Δ* and *csn2Δ* mutant cells, by analysing expression of the silent mating-type reporter gene *mat3-M:ura4*^*+*^. *csn1Δ*/*spd1Δ* and *csn2Δ*/*spd1Δ* double mutants displayed silencing defects similar to those seen in the *csn1Δ* and *csn2Δ* single mutants, confirming that, as for *ddb1Δ*, the observed effects on silencing are independent of Spd1-mediated effects on cell cycle (Figures 5B and 6D). As reported previously, we found that silencing in *ddb1Δ*/*spd1Δ* cells was largely restored upon deletion of Epe1 (Figure 6D). Importantly, Epe1 deletion also restored silencing in *csn1Δ*/*spd1Δ* and *csn2Δ*/*spd1Δ* mutant cells, although to a lesser extent than in *ddb1Δ*/*spd1Δ* cells. Together these observations suggest that the heterochromatin defects observed in *csn1Δ* and *csn2Δ* mutant cells can be partially explained by defects in the regulation of Epe1, likely via the Cul4-Ddb1^Cdt2^ complex. Thus Csn1 and Csn2 appear to contribute to heterochromatin integrity by facilitating the Cul4-Ddb1^Cdt2^-dependent regulation of Epe1. They may also potentially regulate one or more other, as yet unidentified, heterochromatin proteins that are substrates for Cul4-dependent ubiquitin ligase complexes.

## Discussion

In this study we present the first systematic genetic screen for factors required for centromeric heterochromatin integrity in fission yeast. As expected, we identified many factors with known roles in heterochromatin assembly, including RNAi and chromatin modification components. In addition, a further six genes were linked to heterochromatin integrity for the first time, including *stc1*^*+*^, which has been described elsewhere [6]. The remaining five factors could be split into two groups: the COP9 signalosome components Csn1 and Csn2, whose deletion affected silencing at multiple heterochromatic loci, and the splicing-associated proteins Smd3, Saf1 and Saf5, whose absence specifically impaired RNAi-dependent heterochromatin at centromeres. In addition, we have demonstrated that Sde2, a protein previously implicated in heterochromatin integrity, is also functionally linked to splicing, providing novel insights into its molecular function.

Screening for heterochromatin defects via silencing of an embedded *ade6*^*+*^ reporter gene proved an effective strategy, producing only a small number of false positives that were easily identified by secondary screening with an alternative reporter. These mutants presumably have indirect effects on adenine metabolism. The screen was relatively stringent, and it is possible that false negatives may have arisen due to the decision to screen silencing at a single locus, however in general it appears to have been effective in identifying factors with both strong and modest effects on centromeric heterochromatin. In addition, we identified one mutant, a deletion of the nuclear kinase Lsk1, which specifically affected silencing and H3K9 methylation at heterochromatic reporter genes but not at endogenous heterochromatic sequences. Since our focus was on factors with a clear role in maintenance of endogenous heterochromatin, the basis for the specific effect of this mutant on reporter gene silencing was not pursued further. However, given that Lsk1 is required for phosphorylation of RNAPII on Ser2, a modification associated with transcription elongation, one possibility is that deletion of Lsk1 affects production of read-through transcripts important for spreading of silencing from endogenous centromeric repeats to the embedded reporter gene.

### Smd3, Saf1 and Saf5

We have found that cells lacking Smd3, Saf1 or Saf5 all exhibit compromised centromeric heterochromatin associated with a defect in the RNAi pathway. Smd3 is a core snRNP protein involved in splicing, while Saf1 is a splicing-associated factor [42]. Here we identify Saf5 as another protein with physical and functional links to splicing. Both Saf1 and Saf5 were found to associate principally with components of the U5 snRNP and the NineTeen Complex (NTC), suggesting that these factors are likely to be associated with the catalytically activated form of the spliceosome. Connections between RNAi and splicing factors have recently been reported in several systems, including plants [49-53], worms [54-56] and flies [57]. In fission yeast, we have shown previously that conditional mutants of a subset of essential splicing factors disrupt heterochromatin even under permissive conditions, independently of any apparent effects on splicing. These splicing factors were found to associate with pericentromeric chromatin and with the RNAi component Cid12, suggesting a direct role in RNAi recruitment [5]. As was the case for these previously identified splicing factors, the silencing defects in the newly identified splicing mutants are not explained by impaired splicing of the intron-containing RNAi genes *ago1*^*+*^ and *hrr1*^*+*^*.* While we cannot exclude the possibility of other indirect effects, several additional observations support a direct role for splicing-associated factors in promoting processing of centromeric transcripts by the RNAi machinery. First, we have confirmed here previous reports that non-coding RNAs produced from centromeric repeats undergo inefficient splicing, and gone on to show that this splicing is severely impaired in the identified splicing mutants; this demonstrates that these splicing factors act directly on centromeric transcripts. Second, a recent genome-wide study found that at the permissive temperature the conditional splicing mutant *cwf10-1* causes loss of siRNAs and H3K9 methylation at most loci targeted by RNAi, but not at a locus (HOOD 12) where RNAi is known to be recruited via the RNA-binding protein Mmi1; this supports a specific role for this splicing factor in facilitating RNAi at many, but not all, target sites, arguing against the possibility of an indirect effect on RNAi in general [44]. Third, the same study showed that at permissive temperature the *cwf10-1* mutation specifically impairs splicing of cryptic introns but not annotated introns. This was striking since the presence of cryptic splice sites in target transcripts has been shown to increase the efficiency of RNAi in *Cryptococcus* [58]. Although a great deal of further work will be required to elucidate the precise nature of the link between splicing and RNAi, together these observations suggest a model whereby the presence of inefficient splice sites in heterochromatic non-coding RNAs, resulting in either dwelling of the splicing machinery itself, or possibly accumulation of splicing intermediates, may serve as a signal to aid recruitment of the RNAi machinery to target transcripts, possibly via stalling of RNAPII. Interestingly, a phenomenon reminiscent of this has recently been described in *S. cerevisiae* (which lacks RNAi), whereby inefficient splicing appears to act as a regulatory signal flagging transcripts for degradation by the RNA surveillance machinery [59]. This hints at a conserved role for the splicing machinery in the initial identification of target transcripts for processing by downstream regulatory pathways.

Recently, it has been reported that deletion of yet another fission yeast splicing factor, Cwf14, also causes defects in centromeric heterochromatin assembly. However, heterochromatin defects in cells lacking Cwf14 could be partially rescued by replacement of genomic copies of intron-containing RNAi genes (*ago1*^*+*^ and *arb2*^*+*^ or *ers1*^*+*^*)* with intron-less versions, suggesting that the deficiencies in heterochromatin are at least partly due to impaired splicing of mRNA encoding components of the RNAi pathway [60]. This difference between the findings for Cwf14 and the factors described here could potentially result from differences in the RNAi components selected for cDNA rescue experiments. However, the evidence outlined above argues against the possibility that defective splicing of RNAi factors explains heterochromatin defects in all splicing mutants. Rather, it is likely that different splicing factors can impact on RNAi in different ways. The complement of splicing factors required for efficient splicing is known to vary between individual introns [61]. Given that several genes encoding RNAi components contain introns, it is to be expected that many splicing factors, including Cwf14, will be required for splicing of their mRNA. However, other splicing-associated factors, particularly non-essential factors such as those described here, may be largely dispensable for splicing of this set of introns, and instead have a more specific role that directly facilitates processing of heterochromatic transcripts by the RNAi pathway.

### Sde2

Sde2 is a conserved but poorly characterised protein previously shown to be required for genome stability and telomeric silencing [35]. Here we further characterise the effects of Sde2 deletion on the centromere, showing that it causes both de-repression of non-coding centromeric transcripts and a partial reduction in H3K9 methylation, as well as reduced accumulation of siRNAs. Together these phenotypes indicate a defect in processing of centromeric outer repeat transcripts by the RNAi pathway. The molecular function of Sde2 was previously unknown, but we have found that Sde2 physically associates with splicing factors, and, moreover, that deletion of Sde2 results in accumulation of un-spliced transcripts. These observations strongly implicate Sde2 in splicing, and suggest that it may contribute to RNAi-directed chromatin modification in a similar way to other splicing-related factors discussed above. The identification of Sde2 as a splicing-associated factor also sheds new light on its potential role in the maintenance of silencing at the telomeres. In addition to splicing, the spliceosome machinery is involved in 3′ end processing of telomerase RNA (TER1), by promoting RNA cleavage without exon ligation [62]. Deletion of Sde2 might impair this process, resulting in reduced accumulation of mature TER1 and defects in telomere integrity. Alternatively, akin to their role at centromeres, splicing factors such as Sde2 may play a role in promoting the recruitment of additional silencing factors to telomeric transcripts.

### Csn1 and Csn2

We have found that deletions of two of the predicted six components of the fission yeast COP9 signalosome, Csn1 and Csn2, cause defects in heterochromatin. Deletions of two other signalosome components represented in the library, Csn5 and Csn7, did not affect heterochromatin. This is consistent with previous studies that reported UV sensitivity and S-phase delay phenotypes for cells lacking Csn1 or Csn2, but not Csn3, Csn4 or Csn5 [63]. Moreover, while Csn1-5 were all found to be important for the neddylation status of Cullins Cul1 and Cul3 [63,64], only deletion of Csn1, but not Csn4 or Csn5, was found to affect the neddylation status of Cul4 [46]. Together these results suggest a specific role for Csn1 and Csn2 in Cul4 regulation. The S-phase delay and UV sensitivity phenotypes observed upon deletion of Csn1 or Csn2 are very similar to those associated with deletion of the Cul4-associated protein Ddb1, and Csn1, Csn2 and Ddb1 have been reported to function together in the Cul4-dependent regulation of Spd1 [46-48]. Here we show that cells lacking Csn1, Csn2 and Ddb1 also have similar phenotypes with respect to heterochromatin integrity, and that the effects of the *csn* and *ddb1* deletion mutants on silencing are non-additive, suggesting that these factors also function together in the regulation of the heterochromatin regulator Epe1. In support of this, we find that, as for Ddb1, deletion of Csn1 or Csn2 causes increased accumulation of Epe1 at heterochromatic domains, and, moreover, the silencing defects observed in *csn1Δ* and *csn2Δ* mutant cells can be partially rescued by deletion of Epe1. These results suggest that the requirement for Csn1 and Csn2 for heterochromatin integrity can be explained, at least in part, by a role for these proteins in the Cul4-Ddb1^Cdt2^-mediated regulation of Epe1. Epe1 is typically enriched at boundary elements, where it antagonises spreading of heterochromatin into euchromatic regions, and its accumulation within heterochromatin domains (for example as a result of over-expression) is associated with reduced heterochromatin integrity [28,30]. How Epe1 antagonises heterochromatin is unclear, since although it has sequence similarity with histone demethylases, no demethylase activity has been detected *in vitro* [65]. Nonetheless, in combination with previous observations, our findings are consistent with a model in which Epe1 must be depleted from heterochromatin domains in a Csn1/Csn2/Ddb1/Cul4-dependent manner to allow robust heterochromatin assembly to take place. Csn1 and Csn2 might additionally contribute to the function of one or more other Cul4 complexes regulating heterochromatin-related substrates, including, potentially, CLRC. However, since H3K9 methylation levels are no more affected by deletion of Csn1/Csn2 than by deletion of Ddb1, we can rule out a specific requirement for Csn1 and Csn2 in CLRC-mediated H3K9 methylation.

Cullin neddylation promotes activation of CRL E3 ubiquitin ligase activity, and the COP9 signalosome mediates cullin deneddylation [24,66]. Loss of COP9 signalosome activity might therefore be expected to result in increased E3 ubiquitinase ligase activity, and hence increased silencing. The finding that deletion of Csn1 or Csn2 causes a loss of silencing, similar to that seen upon deletion of the CRL component Ddb1, might therefore appear counter-intuitive. However, reduced CRL activity upon COP9 signalosome inactivation has been widely observed, and is thought to occur because Cullin hyper-neddylation results in auto-ubiquitination, and hence destabilisation, of the CRL complex [48,66,67]. Loss of Cul4 deneddylation activity could therefore account for the impaired function of the Cul4-Ddb1^Cdt2^ complex upon deletion of Csn1 or Csn2. However, the lack of dependence on Csn5, which is the subunit harbouring the isopeptidase activity that cleaves Nedd8 from cullins [68], indicates that either a different isopeptidase must be recruited, or an alternative regulatory mechanism is involved. One likely possibility is that Csn1 and Csn2 help to counteract Cul4 hyper-activation by recruiting a deubiquitinase, since the deubiquitinase enzyme Ubp12 has been shown to contribute to signalosome-mediated regulation of Cul1 and Cul3 [67,69]. Another, non-mutually exclusive, possibility is that binding of Csn1 and Csn2 to the CRL may directly stabilise the complex independently of any enzymatic activity [70].

## Conclusions

The genetic screen reported here uncovered several novel factors affecting heterochromatin integrity in fission yeast. We identified known and newly-characterised splicing factors that support processing of heterochromatic transcripts by the RNAi pathway, and elucidated a previously undescribed role for COP9 signalosome components in heterochromatin regulation. The critical bridging protein Stc1 was also identified in this screen and described elsewhere [6]. Our findings shed new light on the regulation of heterochromatin assembly, as well as its integration with other cellular pathways, and provide a more complete understanding of the non-essential factors required for RNAi-directed heterochromatin formation in fission yeast. Any outstanding factors involved in this process likely have either more locus-specific effects, additional roles that render them essential for cell viability, or, like the components described here, facilitatory/regulatory roles in heterochromatin assembly.

## Materials and methods

### Yeast strains and deletion library

A near genome-wide haploid deletion library was constructed and supplied by the Bioneer Corporation and the Korea Research Institute of Biotechnology and Bioscience [36]. The deletion set (v. 2.0) contained 3,088 haploid deletion strains, and we were able to obtain data for 2,660 of these, representing approximately 75% of the non-essential genes of *S. pombe* [38]. Other *S. pombe* strains used are listed in Additional file 1: Table S3. Standard procedures were used for growth and genetic manipulations. Deletion and C-terminal epitope-tagging were achieved by homologous recombination with PCR-amplified fragments containing kanamycin, nourseothicin or hygromycin resistance cassettes flanked by 80 bp of sequence homologous to the insertion site.

### Genetic screening

Manipulations were carried out using a Singer RoToR colony pinning robot, essentially as described previously [37]. First, the library was arrayed in 384 colony format, four colonies per deletion strain, on YES agar containing G418. The tester strain was also arrayed in 384 colony format on YES agar containing ClonNat. Library and tester stain cells were then combined together on ME plates, and incubated at 25°C for 3 days. The resulting cell/spore mixture was then transferred directly onto selective plates (PMG containing G418, ClonNat and cycloheximide, and lacking uracil and leucine) and incubated at 32°C for 5 days. The plates were then incubated at 4°C for 2 days prior to analysis.

### Chromatin and RNA analysis

ChIP was performed as described previously [71], fixing cells in 1% PFA for 15 min at room temperature, and using 1 uL of monoclonal H3K9me2 antibody (m5.1.1) [72] per H3K9me2 ChIP, 1 uL of anti-FLAG M2 antibody (Sigma, F1804) per Epe1-FLAG ChIP, or 5 uL of RNA polymerase II 8WG16 antibody (Covance, MMS-126R) per RNAPII ChIP. Relative enrichments were calculated as the ratio of product of interest to control product (*act1*^+^ or *tRNA*) in IP over input. Northern analysis of centromeric siRNAs and qRT-PCR analysis of transcripts were performed as described previously [5]. qPCR primers, and primers used as siRNA probes, are listed in Additional file 1: Table S4. In all cases histograms represent three biological replicates, and error bars represent one standard deviation.

### Immunoaffinity purification

Immunoaffinity purifications for mass-spec analysis were performed essentially as described previously [5]. Briefly, immunoprecipitations were performed on 5 g of cells using Dynabeads coupled to anti-FLAG M2 antibody (Sigma, F1804) for 15 min. The immunoprecipitated material was treated with 500 U of benzonase, washed, and subjected to on-bead Tryptic digestion prior to preparation for LC-MS/MS analysis. Tables 1 and 2 represent proteins that were identified in three independent purifications of Saf5 or Sde2, respectively, and were not present in control purifications from untagged cells. Ribosomal proteins, which are common contaminants of FLAG purifications, were also excluded.

## Additional files

Additional file 1: Figure S1.
*Cen1:ura4* ^*+*^ silencing assay for mutants not affecting silencing of endogenous centromeric *(dg)* repeats. **Figure S2.** Mating-type locus silencing assay for splicing-associated mutants. **Figure S3.** Genetic interaction assays for splicing-associated mutants. **Figure S4.** Centromeric silencing assay for splicing-associated mutants bearing cDNA versions of *ago1* ^*+*^ and *hrr1* ^*+*^. **Figure S5.** Sequence data for the intron in centromeric (*dg*) sequence. **Table S1.** Screen data for all genes with annotated roles in chromatin silencing at centromeres. **Table S2.** Details of screen hits without annotated roles in chromatin silencing at centromeres. **Table S3.** Yeast strains used in this study. **Table S4.** Primers used in this study. Additional file 2:
**Screen results for all deletion strains represented in the library.**

## Abbreviations

CLRC: Clr4 complex
CRL: Cullin-RING ubiquitin ligase
DCAF: DDB1 and Cul4-associated factor
HDAC: histone deacetylase
H3K9me: methylation of histone H3 on lysine 9
ORF: open reading frame
PCR: polymerase chain reaction
qRT-PCR: quantitative reverse transcription-PCR
RDRC: RNA-dependent RNA polymerase complex
RITS complex: RNA-induced transcriptional silencing complex
RNAi: RNA interference
RNAPII: RNA polymerase II
SHREC: Snf2/HDAC-containing Repressor Complex
snRNP: small nuclear ribonucleic particle
