# Supplementary material for: A systematic genetic screen identifies new factors influencing centromeric heterochromatin integrity in fission yeast
Source: Genome Biol. 2014 Oct 2;15(10):481. doi: 10.1186/s13059-014-0481-4 (PMC4210515; doi:10.1186/s13059-014-0481-4)
Supplement: Additional file 1: Figure S1. — Cen1:ura4 + silencing assay for mutants not affecting silencing of endogenous centromeric (dg) repeats. Figure S2. Mating-type locus silencing assay for splicing-associated mutants. Figure S3. Genetic interaction assays for splicing-associated mutants. Figure S4. Centromeric silencing assay for splicing-associated mutants bearing cDNA versions of ago1 + and hrr1 +. Figure S5. Sequence data for the intron in centromeric (dg) sequence. Table S1. Screen data for all genes with annotated roles in chromatin silencing at centromeres. Table S2. Details of screen hits without annotated roles in chromatin silencing at centromeres. Table S3. Yeast strains used in this study. Table S4. Primers used in this study. [file 13059_2014_481_MOESM1_ESM.pdf]

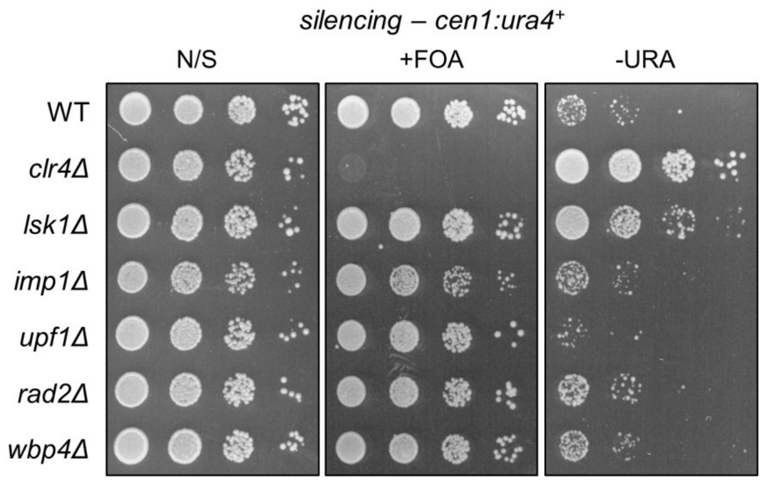

Figure S1. **Of five mutants affecting silencing at *cen1:ade6*$^{+}$ but not endogenous centromeric (*dg*) repeats, only *lsk1Δ* also affects silencing of *cen1:ura4*$^{+}$*.*** Silencing of a *cen1:ura4*$^{+}$ reporter gene was assessed by spotting serial dilutions of the indicated strains on non-selective plates (N/S), plates lacking uracil (-URA) and plates supplemented with FOA (+FOA).

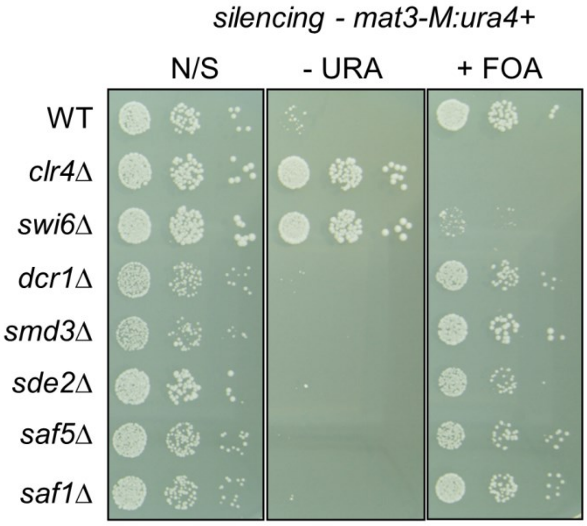

Figure S2. Splicing-associated mutants do not affect silencing at the mating-type locus. Silencing of a *ura4*$^{+}$ reporter gene inserted into the silent mating-type locus (*MAT3-M:ura4*$^{+}$) was assessed by spotting serial deletions of the indicated strains on non-selective plates (N/S), plates lacking uracil (-URA) and plates supplemented with FOA (+FOA). Loss of silencing results on growth on –URA and loss of resistance to FOA.

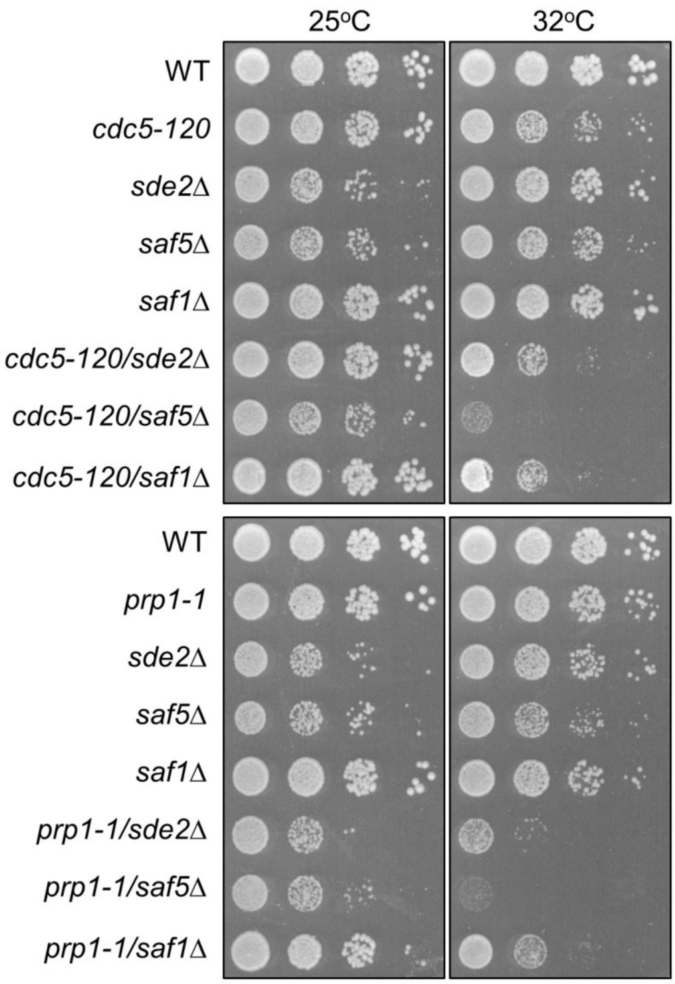

Figure S3. *sde2Δ*, *saf5Δ* and *saf1Δ* exhibit negative genetic interactions with known splicing mutants *cdc5-120* and *prp1-1*. Equivalent cell numbers of the indicated strains were spotted in serial dilutions and incubated at the indicated temperatures for 3-5 days.

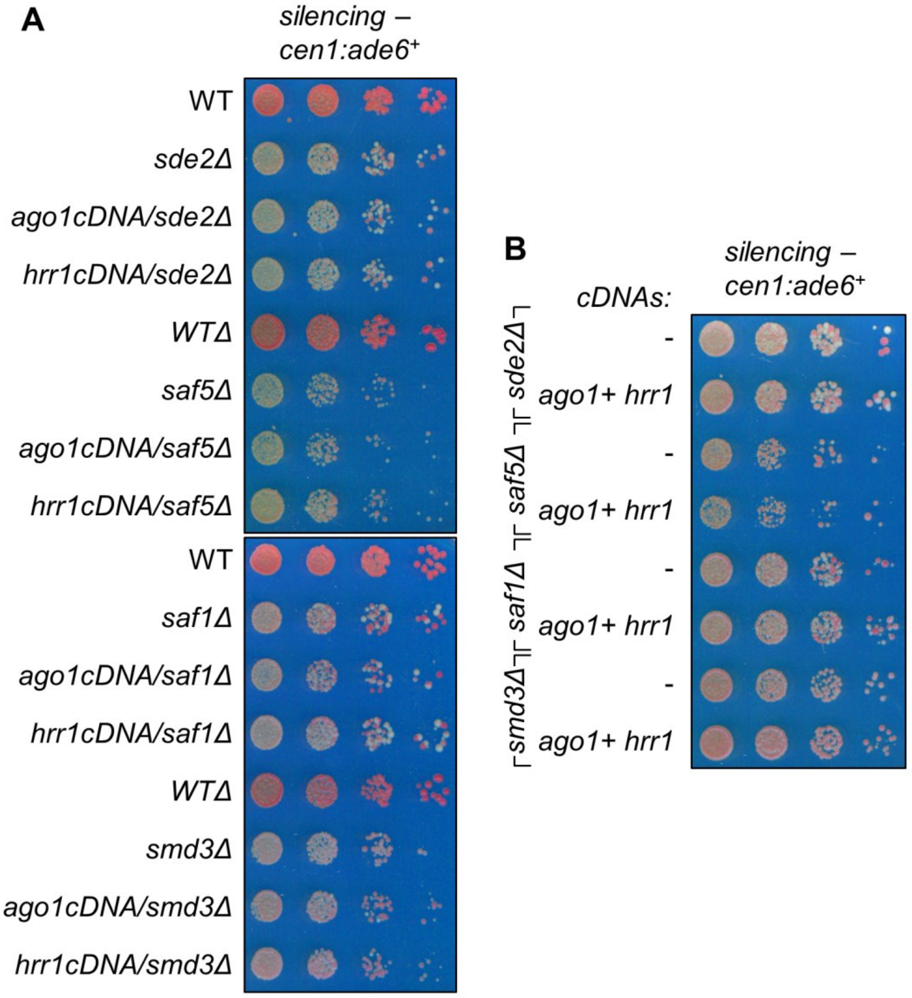

Figure S4. Silencing defects in splicing mutants are not explained defects in splicing of RNAi components Ago1 and Hrr1. A *cen1:ade6*$^{+}$ silencing assay was performed on splicing mutant cells in which genomic copies of intron-containing RNAi factor genes *ago1*$^{+}$ and *hrr1*$^{+}$ had been replaced by cDNA versions either singly (A), or in combination (B). Equivalent cell numbers were spotted in serial dilutions on plates containing 10ug/ml adenine.

A

```
CTTTTTCCATCCGCAGTTGGGAGTACATCATTCCTACTTC
GATATACTTTTGAAAGAGAAAAGTGAACCGATTGGAATTG
TTTCGACAACACGAATTTTACTTCTAGGTCATATATTTAT
GGCGATGCCAAACAACAATATTGTTGAGACAgtaagttaa
gcaaatggctaaataaatccaagactgttgttgagtgctg
tggatctcaatattatcagtatctactcttctcgatgatc
ctgtaaattttgttatctttttttatttgcagttactga
taatactagCATTTTGCATGCTTTTTTTTATTTGGATGTT
CTTCCAATTGATCAATACAGTAACTTTAGAACTATCATCG
CTATGCCTTTAAATGACCGCACTAAAAGCATGGTACCAAA
```

B

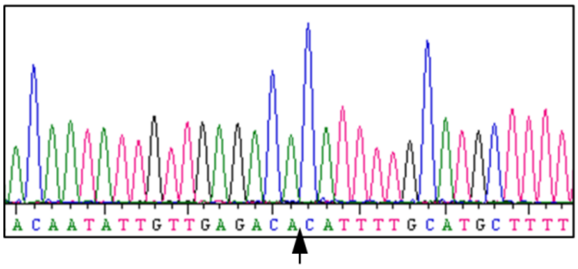

Figure S5. Centromeric (dg) transcripts undergo splicing. (A) DNA sequence of the intron-containing region of *cen-dg*. Arrows indicate the location of the primers used for RT-PCR analysis; the intron is indicated in blue, and 5' and 3' splice sites are underlined. (B) Sequencing data for the lower band observed in Figure 4C, confirming that it represents a bona fide splice product. The arrow indicates where the intron has been removed.

Table S1. Screen results for all genes with annotated roles in chromatin silencing at centromeres according to PomBase. Silencing of the *cen1:ade6*$^{+}$ reporter was scored via colony colour on low adenine media (colour score), and colony growth on media lacking adenine (growth score). A score of 4 is equivalent to wild-type silencing, and 1 represents strong de-repression. Indicated are genes detected in the screen, not detected, incorrectly deleted (and therefore not detected), and absent from the library. 'c' denotes genes involved in silencing at centromere central core but not outer repeats.

	Systematic name	Gene name	Colour score	Growth score
detected	SPAC664.01c	*swi6*	1	1
	SPBC428.08c	*clr4*	1	1
	SPCC11E10.08	*rik1*	1	1
	SPCC613.12c	*raf1*	1	1
	SPCC970.07c	*raf2*	1	1
	SPAC18G6.02c	*chp1*	1	1
	SPAC140.03	*arb1*	1	1
	SPAC13G7.07	*arb2*	1	1
	SPCC1393.05	*ers1*	1	1
	SPBP8B7.28c	*stc1*	1	1
	SPCC663.12	*cid12*	1	1
	SPAC6F12.09	*rdp1*	2	1
	SPAC31G5.18c	*sde2*	3	1
	SPCC188.13c	*dcr1*	1	2
	SPAC1783.05	*hrp1*	1	2
	SPBC16D10.07c	*sir2*	2	2
	SPBC31F10.13c	*hip1*	2	2
	SPBC609.05	*pob3*	2	2
	SPCC132.02	*hst2*	4	2
not detected	SPBC342.06c	*rtt109*	3	4
	SPBC947.08c	*hip4*	4	4
	SPBC800.03	*clr3*	4	4
	SPAC1B3.17	*clr2*	4	4
	SPBC2D10.17	*clr1*	4	4
	SPBP35G2.10	*mit1*	4	4
incorrect	SPCC736.11	*ago1*	3	4
	SPCC1739.03	*hrr1*	3	4
	SPBC365.06	*pmt3*	3	4
absent (non-essential)	SPBC83.03c	*tas3*	-	-
	SPAC3A11.08	*cul4*	-	-
	SPBC16C6.10	*chp2*	-	-
	SPAC1783.04c	*hst4*	-	-
	SPBC31F10.14c	*hip3*	-	-
	SPBC15D4.03	*slm9*	-	-
	SPBC14F5.12c	*cbh2*	-	-
	SPAC9E9.10c	*cbh1*	-	-
	SPBC1105.04c	*cbp1*	-	-
	SPAC1F7.01c	*spt6*	-	-
	SPBC577.15c	*sim3* c	-	-
	SPBC19G7.16	*iws1*	-	-
	SPBC21D10.12	*hob1*	-	-
absent (essential)	SPBC36.05c	*clr6*	-	-
	SPBC12C2.10c	*pst1*	-	-
	SPBP19A11.06	*lid2*	-	-
	SPBC1105.17	*cnp1* c	-	-
	SPBC18E5.03c	*sim4* c	-	-
	SPAC25B8.14	*mal2* c	-	-
	SPBC16C6.12c	*las1*	-	-
	SPCC4G3.18	*rix1*	-	-
	SPAC13G7.08c	*crb3*	-	-
	SPCC830.03	*grc3*	-	-
	SPBP23A10.08	*alp5*	-	-
	SPAC22E12.07	*rna1*	-	-

Table S2. Screen hits without annotated roles in chromatin silencing at centromeres (according to PomBase). Silencing of the *cen1:ade6*$^{+}$ reporter was scored via colony colour on low adenine media (colour score), and colony growth on media lacking adenine (growth score). A score of 4 is equivalent to wild-type silencing, and 1 represents strong de-repression. Strains in which the designated gene proved not to be deleted, and strains that did not exhibit de-repression of endogenous heterochromatic transcripts, were not analysed further.

Systematic name	Gene name	Colour score	Growth score	Correct deletion	Elevated heterochromatic transcripts
SPBC19C2.14	*smd3*	2	1	yes	yes
SPAC1610.01	*saf5*	2	1	yes	yes
SPAC17H9.10c	*ddb1*	2	2	yes	yes
SPBC215.03c	*csn1*	2	2	yes	yes
SPAPB17E12.04c	*csn2*	2	2	yes	yes
SPCC663.11	*saf1*	3	2	yes	yes
SPAC17G8.05	*med20*	4	2	yes	yes
SPAC2F3.15	*lsk1*	2	1	yes	no
SPBC18H10.07		3	2	yes	no
SPBC1604.08c	*imp1*	4	2	yes	no
SPAC16C9.06c	*upf1*	4	2	yes	no
SPAC3G6.06c	*rad2*	4	2	yes	no
SPCC61.02	*spt3*	1	1	no	-
SPBC1539.03c		1	2	no	-

Table S3. Strain List

Strain	Genotype
FY11630	*h- sph:ade6-lys1-NAT cyclohexamideR [ade6-210 linked to ura4+] ura4-D18 leu1-32 (mat1M mat2/3::LEU2^{+})*
FY1180	*h+ ade6-210 leu1-32 ura4-D18 otr1R(Sph1):ade6+*
FY8897	*h- clr4Δ::kanR ade6-210 leu1-32 ura4-D18 lys1:Nat otr1R(Sph1):ade6+*
FY7005	*h+ dcr1Δ::natR ade6-210 leu1-32 ura4-D18 otr1R(Sph1):ade6+*
EB139	*h+ sir2Δ::natR ade6-210 leu1-32 ura4-D18 otr1R(Sph1):ade6+*
EB920	*h+ med20Δ::natR ade6-210 leu1-32 ura4-D18 otr1R(Sph1):ade6+*
EB780	*h- csn1Δ::kanR ade6-210 leu1-32 ura4-D18 otr1R(Sph1):ade6+*
EB499	*h- csn2Δ::kanR ade6-210 leu1-32 ura4-D18 otr1R(Sph1):ade6+*
EB776	*h- ddb1Δ::kanR ade6-210 leu1-32 ura4-D18 otr1R(Sph1):ade6*
EB981	*h+ sde2Δ::kanR ade6-210 leu1-32 ura4-D18 otr1R(Sph1):ade6+*
EB546	*h+ saf5Δ::KanR ade6-210 leu1-32 ura4-D18 otr1R(Sph1):ade6+*
EB531	*h+ saf1Δ::kanR ade6-210 leu1-32 ura4-D18 otr1R(Sph1):ade6+*
EB785	*h+ smd3Δ::kanR ade6-210 leu1-32 ura4-D18 otr1R(Sph1):ade6+*
EB530	*h+ lsk1Δ::kanR ade6-210 leu1-32 ura4-D18 otr1R(Sph1):ade6+*
FY8684	*h+ ade6-DN/N leu1-32 ura4-D18/DSE arg3D4 otr1R(Sph1):ade6+*
FY9640	*h+ clr4Δ::ura4+ ade6-DN/N leu1-32 ura4-D18/DSE otr1R(Sph1):ade6+*
EB2221	*lsk1Δ::kanR ade6-DN/N leu1-32 ura4-D18/DSE otr1R(Sph1):ade6+*
FY9024	*h- rik1Δ::kanR ade6-210 leu1-32 ura4-D18 lys1:Nat otr1R(Sph1):ade6+*
EB553	*h+ saf5-3xFLAG-natR ade6-210 leu1-32 ura4-D18 otr1R(Sph1):ade6+*
EB901	*h+ sde2-3xFLAG-natR ade6-210 leu1-32 ura4-D18 otr1R(Sph1):ade6+*
EB1906	*h+ cwf11::hygR ade6-216 leu1-32 ura4-D18*
EB2131	*cwf11::hygR sde2Δ::kanR ade6-210/216 leu1-32 ura4-D18*
EB2132	*cwf11::hygR saf5Δ::kanR ade6-210/216 leu1-32 ura4-D18*
EB2133	*cwf11::hygR saf1Δ::kanR ade6-210/216 leu1-32 ura4-D18*
EB1744	*sde2Δ::kanR dcr1Δ::natR ade6-210 leu1-32 ura4-D18 otr1R(Sph1):ade6+*
EB1745	*saf5Δ::kanR dcr1Δ::natR ade6-210 leu1-32 ura4-D18 otr1R(Sph1):ade6+*
EB1743	*saf1Δ::kanR dcr1Δ::natR ade6-210 leu1-32 ura4-D18 otr1R(Sph1):ade6+*
FY511	*h90 ade6-216 leu1-32 ura4-D18 mat3-M:ura4^{+}*
EB830	*h90 csn2Δ::kanR ade6-216 leu1-32 ura4-D18 mat3-M:ura4^{+}*

EB831	*h90 ddb1Δ::kanR ade6-216 leu1-32 ura4-D18 mat3-M:ura4*$^+$
EB832	*h90 csn1Δ::kanR ade6-216 leu1-32 ura4-D18 mat3-M:ura4*$^+$
FY7429	*h90 clr4Δ::leu2 ade6-210 leu1-32 ura4-D18 mat3:ura4*$^+$
EB2227	*csn1Δ::kanR ddb1Δ::natR ade6-210 leu1-32 ura4-D18 otr1R(Sph1):ade6+*
EB2228	*csn2Δ::kanR ddb1Δ::natR ade6-210 leu1-32 ura4-D18 otr1R(Sph1):ade6+*
EB2229	*csn1Δ::kanR ddb1Δ::natR ade6-210 leu1-32 ura4-D18 mat3-M:ura4*$^+$
EB2230	*csn2Δ::kanR ddb1Δ::natR ade6-210 leu1-32 ura4-D18 mat3-M:ura4*$^+$
EB1000	*h-smt0 ade6-M210 leu1-32 ura4-D18 epe1:CBP-FLAG-NatR*
EB1308	*h+ epe1:CBP-FLAG-NatR csn1::kanR spd1::hygR leu1-32 ura4-D18 ade6-DN/N otr1(Sph):ade6+*
EB1310	*h+ epe1:CBP-FLAG-NatR csn2::kanR spd1::hygR leu1-32 ura4-D18 ade6-DN/N otr1(Sph):ade6+*
EB1312	*h+ epe1:CBP-FLAG-kanR ddb1::natR spd1::hygR leu1-32 ura4-D18 ade6-DN/N otr1(Sph):ade6+*
EB730	*h90 mat3::ura4+ epe1Δ::leu+ leu1-32 ade6-210? Ura4-D18*
EB1558	*h90 csn1Δ::kanR spd1Δ::hygR leu1-32 ade6-210 Ura4-D18 mat3:ura4+*
EB1559	*h90 csn1Δ::kanR spd1Δ::hygR epe1Δ::leu+ leu1-32 ade6-210 uraD18 mat3:ura4+*
EB1560	*h90 csn2Δ::kanR spd1Δ::hygR leu1-32 ade6-210 Ura4-D18 mat3:ura4+*
EB1561	*h90 csn2Δ::kanR spd1Δ::hygR epe1Δ::leu+ leu1-32 ade6-210 uraD18 mat3:ura4+*
EB1562	*h90 ddb1Δ::natR spd1Δ::hygR leu1-32 ade6-210 Ura4-D18 mat3:ura4+*
EB1563	*h90 ddb1Δ::natR spd1Δ::hygR epe1Δ::leu+ leu1-32 ade6-210 uraD18 mat3:ura4+*
EB2222	*h+ lsk1Δ::kanR ade6-210 leu1-32 ura4-D18 otr1R(Sph1):ura4*$^+$
EB2223	*h+ imp1Δ::kanR ade6-210 leu1-32 ura4-D18 otr1R(Sph1):ura4*$^+$
EB2224	*h+ upf1Δ::kanR ade6-210 leu1-32 ura4-D18 otr1R(Sph1):ura4*$^+$
EB2225	*h+ rad2Δ::kanR ade6-210 leu1-32 ura4-D18 otr1R(Sph1):ura4*$^+$
EB2226	*h+ wbp4Δ::kanR ade6-210 leu1-32 ura4-D18 otr1R(Sph1):ura4*$^+$
FY9296	*h90 swi6Δ::kanR ade6-210 leu1-32 ura4-D18 mat3:ura4*$^+$
FY9299	*h90 dcr1Δ::natR ade6-210 leu1-32 ura4-D18 mat3:ura4*$^+$
EB2207	*h90 smd3Δ::kanR ade6-210/216 leu1-32 ura4-D18 mat3:ura4*$^+$
EB2208	*h90 sde2Δ::kanR ade6-210/216 leu1-32 ura4-D18 mat3:ura4*$^+$
FY6231	*h90 saf5Δ::kanR ade6-210/216 leu1-32 ura4-D18 mat3:ura4*$^+$
FY6230	*h90 saf1Δ::kanR ade6-210/216 leu1-32 ura4-D18 mat3:ura4*$^+$
FY8995	*h+ cdc5-120 ade6-210 leu1-32 ura4-D18 lys1:Nat otr1R(Sph1):ade6+*
EB2215	*sde2Δ::kanR cdc5-120 ade6-210 leu1-32 ura4-D18 otr1R(Sph1):ade6+*
EB2216	*saf5Δ::kanR cdc5-120 ade6-210 leu1-32 ura4-D18 otr1R(Sph1):ade6+*

EB2217	*saf1Δ::kanR cdc5-120 ade6-210 leu1-32 ura4-D18 otr1R(Sph1):ade6+*
FY7478	*h+ prp1-1 ade6-210 leu1-32 ura4-D18 arg3-D4 his3D1*
EB2218	*sde2Δ::kanR prp1-1 ade6-210 leu1-32 ura4-D18*
EB2219	*saf5Δ::kanR prp1-1 ade6-210 leu1-32 ura4-D18*
EB2220	*saf1Δ::kanR prp1-1 ade6-210 leu1-32 ura4-D18*
EB763	*sde2Δ::kanR ago1::ago1cDNA ade6-210 leu1-32 ura4-D18 otr1R(Sph1):ade6+*
EB768	*sde2Δ::kanR hrr1::hrr1cDNA ade6-210 leu1-32 ura4-D18 otr1R(Sph1):ade6+*
EB2211	*sde2Δ::kanR ago1::ago1cDNA hrr1::hrr1cDNA ade6-210 leu1-32 ura4-D18 otr1R(Sph1):ade6+*
EB764	*saf5Δ::KanR ago1::ago1cDNA ade6-210 leu1-32 ura4-D18 otr1R(Sph1):ade6+*
EB769	*saf5Δ::KanR hrr1::hrr1cDNA ade6-210 leu1-32 ura4-D18 otr1R(Sph1):ade6+*
EB2212	*saf5Δ::KanR ago1::ago1cDNA hrr1::hrr1cDNA ade6-210 leu1-32 ura4-D18 otr1R(Sph1):ade6+*
EB765	*saf1Δ::kanR ago1::ago1cDNA ade6-210 leu1-32 ura4-D18 otr1R(Sph1):ade6+*
EB770	*saf1Δ::kanR hrr1::hrr1cDNA ade6-210 leu1-32 ura4-D18 otr1R(Sph1):ade6+*
EB2213	*saf1Δ::kanR ago1::ago1cDNA hrr1::hrr1cDNA ade6-210 leu1-32 ura4-D18 otr1R(Sph1):ade6+*
EB2209	*smd3Δ::kanR ago1::ago1cDNA ade6-210 leu1-32 ura4-D18 otr1R(Sph1):ade6+*
EB2210	*smd3Δ::kanR hrr1::hrr1cDNA ade6-210 leu1-32 ura4-D18 otr1R(Sph1):ade6+*
EB2214	*smd3Δ::kanR ago1::ago1cDNA hrr1::hrr1cDNA ade6-210 leu1-32 ura4-D18 otr1R(Sph1):ade6+*

Table S4. Primer List

**qRT-PCR**	
q_cen(dg)_FOR	AATTGTGGTGGTGTGGTAATAC
q_cen(dg)_REV	GGGTTCATCGTTTCCATTCAG
q_mat_FOR	GTCCGAGGCAATACAACTTTGG
q_mat_REV	GGTTGACAGTAGGAGATATTTACAG
q_ade6_FOR	ATGCTTATCCTACAACTGAGACC
q_ade6_REV	TGAATTGAGAAGGGAAGACGAG
q_act_FOR	GGTTTCGCTGGAGATGATG
q_act_REV	TGAATTGAGAAGGGAAGACGAG
q_tRNAGly_Fw	AATGCTTTGGCCGGGAATCGAA
q_tRNAGly_Rv	CATTAAGCTTTGGTGGTTTAG
nda2_intron1F	TGTATACTCTAAATAGTTGGCAATCG
nda2_ intron1R	GCCATGCTCCAAACAGTACA
nda2_exon2F	TTTTCTGAAACCGGGCAAG
nda2_exon2R	AACGTTAGGCTCAAGATCAACA
nda3_intron1F	AATATTCAAAGCTAATCAATAGGTTCA
nda3_intron1R	GACTTGGTTTCCGCATTGAC
nda3_exon4F	TGCTGTTTTGGACGTTGTTC
nda3_exon4R	ACCACCACCCAAAGAGTGAG
**RT-PCR**	
dg-1	CCCATCCGCAGTTGGGAG
dg-2	TACCATGCTTTTAGTGCGG
**siRNA probes**	
IK8	ATTCCTTTCTGAACCTCTCTGTTAT
IK9	TTTGATGCCCATGTTCATTCCACTTG
IK10	GGGAGTACATCATTCCTACTTCGATA
snR58	GATGAAATTCAGAAGTCTAGCATC
